# Protective Effects of Foam Rolling against Inflammation and Notexin Induced Muscle Damage in Rats

**DOI:** 10.7150/ijms.37981

**Published:** 2020-01-01

**Authors:** Ana Pablos, Diego Ceca, Adrián Jorda, Pilar Rivera, Carlos Colmena, Laura Elvira, Francisco M. Martínez-Arnau, Soraya L. Valles

**Affiliations:** 1 Faculty of Physical Activity and Sport Sciences, Universidad Católica de Valencia San Vicente Mártir, Valencia, Spain; 2 Department of Education, Universidad Internacional de Valencia, Valencia, Spain; 3 Department of Physiology, School of Medicine, University of Valencia, Valencia, Spain; 4 Faculty of Nursing, Universidad Católica de Valencia San Vicente Mártir, Valencia, Spain; 5 Departament of Physiotherapy, University of Valencia, Valencia, Spain

**Keywords:** foam rolling, muscle recovery, inflammation, cell death, performance

## Abstract

It is known that high-intensity exercise can cause inflammation and damage in muscle tissue, and in recent years, physical therapists and fitness professionals have begun to use foam rolling as a recovery method to improve performance. Despite the lack of basic science studies to support or refute the efficacy of foam rolling, the technique is very widely used in the sports world. In this respect, we investigated whether foam rolling could attenuate muscle damage and inflammation. Female Wistar rats were assigned to control (C), foam rolling (FR), notexin without foam rolling (N) and notexin with foam rolling (NFR) groups. A 4.5 x 2 cm foam roller was used to massage their hind legs (two 60-second repetitions twice a day for 3 days). Motor function tests (Balance Beam Test and Grip strength) were used. We detected an increase in time and foot faults when crossing a beam in the N group compared to C and FR rats. In contrast, a significant decrease was detected in both tests in NFR compared to N rats. Muscle power was measured with a grip strength test and better performance was detected in NFR rats compared to N rats. Furthermore, an increase of pro-inflammatory proteins was noted in the N group, while there was a decrease in the NFR group. On the contrary, an increase in PPAR-γ (anti-inflammatory protein) in the NFR group compared to the N group demonstrates the anti-inflammatory properties of the foam rolling technique. In summary, applying foam rolling after damage has benefits such as an increase in anti-inflammatory proteins and a reduction of pro-inflammatory proteins, resulting in muscle recovery and better performance.

## Introduction

It is known that high-intensity exercise can cause inflammation and damage in muscle tissue, altering immune function^1^. In the inflammatory process, cytokines, pro- and anti-inflammatory proteins, phagocytes and leukocytes act as recovery and regeneration substances after muscle injury and some of them, such as interleukin 1 β (IL-1β) and tumour necrosis factor α, initiate the inflammatory and repair process^2^. Moreover, anti-inflammatory proteins, such as PPAR-γ can suppress inflammatory processes^3^,^4^. It is not uncommon to think that when changes or failures occur in their mechanisms of action, they can have fatal consequences for our organism. The inflammation originates thanks to a set of immune cells involved in the process that through signalling pathways composed of different groups of pro and anti-inflammatory molecules cause different changes in the inflamed area. The resolution of the inflammatory process happens after the neutralization of the trigger. From this moment on, part of the cells of the immune system also begin to generate an anti-inflammatory activity, including lipoxins (for example, LXA4, RvE1) and cytokines (such as, IL-10, IL-37, TGF-β)^5^. Acute inflammatory processes will be resolved relatively quickly, while, however, resolution processes are not achieved in chronic inflammation. The differences between both types of inflammation, acute and chronic, reside at different levels. With respect to the cells that intervene in acute inflammation, neutrophils intervene in the context of an infection and eosinophils and mast cells in the case of allergies. The chemical mediators involved in acute inflammation would be the complement system, the kinins, the prostaglandins, the leukotrienes, the cytokines coming from several immune cells and the gamma interferon of the T lymphocytes. On the other hand, in chronic inflammation we would have the participation of macrophages and lymphocytes mainly, which would produce cytokines as main chemical mediators of this type of inflammation.

In recent years, physical therapists and fitness professionals have begun to use foam rolling as a recovery method to improve performance. Foam rolling is a commonly used technique that requires individuals to use their own body mass on a foam roller to apply pressure to the soft tissue^6^.

Different studies in which foam rolling has been used after performing physical tests or applying a delayed-onset muscle soreness induction protocol have shown that the use of this instrument makes it possible to reduce perceived fatigue and muscle pain^7^,^8^, concluding that it is an effective technique for muscle recovery^9^.

Despite this, there is still little evidence regarding the influence of foam rolling at a physiological level that can explain the aforementioned effects. Specifically, some authors have observed that after applying the technique there was a decrease in pulse wave velocity and an increase in the concentration of nitric oxide, concluding that it could provide benefits in terms of arterial function^10^. On the other hand, recent data have shown that an increase in blood flow was achieved after foam rolling^11^, and blood circulation has been shown to have an essential role in tissue healing and therefore in muscle recovery^12^. It has also been proved that foam roller application is connected to increased muscle oxygen saturation, thus promoting recovery^8^.

Despite the lack of basic science studies to support or refute the efficacy of foam rolling, the technique is very widely used in the sports world. In this respect, the present study aims to further explore the effects of foam rolling on muscle damage and inflammation. An experimental design was carried out applying foam roller technique during 3 days after Notexin-induced injury. Notexin is a presynaptic phospholipase A_2_ neurotoxin isolated from snake venom, has been described as causing complete destruction and rapid regeneration of muscle fibers and providing an effective and reliable model of skeletal muscle injury/regeneration in the mouse^13^. The degeneration caused by notexin involve Ca^2+^ overload and activation of Ca^2+^-dependent proteases^13^.

## Methods

### Design

This study tested the hypothesis that the application of foam rolling after notexin-induced muscle damage could act as a protective technique, so we studied its effects on rat muscle, looking at inflammation, changes in cell death and changes in exercise performance with the samples obtained after 6 training sessions.

### Subjects: Animals

Twenty female Wistar rats (weight 200-250 g; age 7 months) were randomly divided into 4 groups with 5 in each group, including: control (C), foam rolling (FR), notexin without foam rolling (N) and notexin with foam rolling (NFR). When notexin was used, only one leg was injected.

All the rats were fed ad libitum on a standard diet and were kept on a 12-h light/12-h dark cycle, keeping the room temperature at 22ºC. The rats were numb before the injection with notexin, the blood draw and the application of a foam roller with inhalation anesthesia (5% inofluoran by induction and 2% maintenance). At the end of the study, the animals were euthanized (pentobarbital 50 mg/Kg) for removal of the quadriceps muscle. Death certification was due to heartbeat and stiffness. For the experiments, the estral phase chosen was diestro. It was identified by vaginal lavage and the appearance of rounded cells of small size and with very few keratinized cells.

All animal procedures were carried out in accordance with the European legislation on the use and care of laboratory animals (EEC 86/609). Experimental research on rats was performed with the approval of the ethics committee on animal research of the University of Valencia, Spain (Code: 2018/VSC/PEA/0004).

### Foam Rolling Program

A high-density foam roller was applied to the hind legs of 10 rats (FR and NFR groups). The massage was applied by the veterinarian in two 60-second repetitions, twice a day (at 10 a.m. and 5 p.m.) for 3 days. The pressure applied was 25% of the rat's weight. To know the pressure exerted, rats were weighted before and during the application of foam roller. The measures of the foam roller were 4.5 x 2 cm.

To induce muscle injury, 200 µl of notexin was injected intramuscularly at 10 µg/ml in the quadriceps, only in one leg of the rat. Notexin was injected 24 h before foam roller application. The control rats were injected with 200 µl of saline solution.

### Determination of cytokines IL-1 and TNF-α

Plasma was obtained from the rats and used to determine IL-1β and TNF-α concentration (pg/ml) using ELISA kits (Pierce Biotechnology, Inc.) (Catalogue number. IL-1β: ER2L1B. TNFα: ER3TNFA). The blood was obtained from the lateral vein of the tail (1 ml) prior anaesthesia. Plasma was obtained and for each test 100 µl was used.

### Western blot analysis

The protein extracts from the quadriceps were mixed with equal volumes of SDS buffer (0.125 M Tris-HCl, pH 6.8, 2% SDS, 0.5% (v/v) 2-mercaptoethanol, 1% bromophenol blue and 19% glycerol) and then boiled for 5 min. The protein concentration was determined using a modified Lowry method^14^. The proteins were separated by SDS-PAGE gels and transferred to nitrocellulose membranes using standard techniques. The membranes were blocked with 5% dried milk in TBS containing 0.05% Tween-20 and then incubated with the corresponding antibodies following the manufacturer's recommendations. The blots were washed three times with a washing buffer (phosphate-buffered saline, 0.2% Tween 20) for 15 min each and then incubated for 1 h with a horseradish peroxidase-linked secondary anti-rabbit or anti-mouse IgG antibody (Cell Signaling Technologies, Barcelona, Spain). As above, the blots were washed three times and developed using the enhanced chemiluminescence (ECL) procedure as specified by the manufacturer (Pharmacia biotechnology, San Francisco, CA, USA). Auto-radiographic signals were assessed using a Bio-Rad scanning densitometer. Anti-Cytochrome c (anti-Cyt c) (1:500) (PA5-51550), anti-VEGF (1:1000) (MA-5-13182), anti-COX-2 (1:500) (PA5-17614), anti-Smac/Diablo (1:500) (MA1-936) and anti-peroxisome proliferator-activated receptor antibody (PPAR-γ) (1:250) (MA5-14889) were obtained from Thermo Fisher Scientific. Monoclonal anti-apoptosis-inducing factors (anti-AIF) (1:500) (PR523001) were obtained from Sigma-Aldrich Biotech (Barcelona, Spain). Anti-p38 (1:500) (sc-81621), anti-p-p38 (1:500) (sc-7973), anti-STAT (1:1000) (sc-293151), anti-NF-ᴋB (1:1000) (sc-515045), anti-IᴋB (1:500) (sc-373893) and anti-α-tubulin (sc-5286) monoclonal antibodies were acquired from Santa Cruz Biotechnology (Barcelona, Spain).

The tubulin protein was used as control. Loading controls are essential for proper interpretation of western blots and can be used to normalize the levels of protein detected by confirming that protein loading is the same across the gel. All other reagents are of analytical or culture grade purity.

### Motor function

Motor function was determined by two different tests: Balance Beam Test and Grip Strength; in the first test, the rats' ability to pass along a narrow beam to reach a dark box was evaluated to assess the motor coordination of animals. To force the rats to pass along the beam, a white light illuminated the beginning of the beam. The wooden beam (1 x 150 cm) was elevated to a height of 1.5 m above the floor. The time required crossing to the escape box at the other end of beam and the number of forelimb and hind-limb paw slips were recorded. A paw slip was defined as any paw coming off the top of the beam or any limb use on the side of the beam. On the day of the test, four trials were performed before recording the results. The goal was to familiarize the rats with the beam and to train them in the presence of the dark box at the end of the beam. The narrow beam and the dark box were cleaned after each trial with ethanol.

In a second type of test, grip strength was measured non-invasively by taking advantage of the rat's instinctive tendency to grab as they are gently pulled backward. This method can be used to test for limb grip using a horizontal bar with a small diameter (0.1 cm), which is attached to a force transducer. The length of time the rat was able to hold the bar was recorded. This latency to grip loss is considered as an indirect measure of grip strength^15^ and the cut-off time was taken as 60 seconds.

The rat was allowed to hold, with the forepaws, a steel wire (2 mm in diameter and 80 cm in length), placed at a height of 50 cm over a cushion support. The length of time the rat was able to hold the wire was recorded. This latency to grip loss is considered as an indirect measure of grip strength^15^, and the cut-off time was taken as 90 seconds.

### Statistical analyses

Statistical analyses were performed using the Statistical Package for the Social Sciences (SPSS Inc., Chicago, IL). The data was analysed by parametric ANOVA (there is homogeneity of the variances). In addition to this, Scheffe method was used to determine post-hoc pair-wise comparisons. A p-value less than 0.05 were considered to indicate statistical significance.

## Results

### Exercise performance

**Figure 1:**
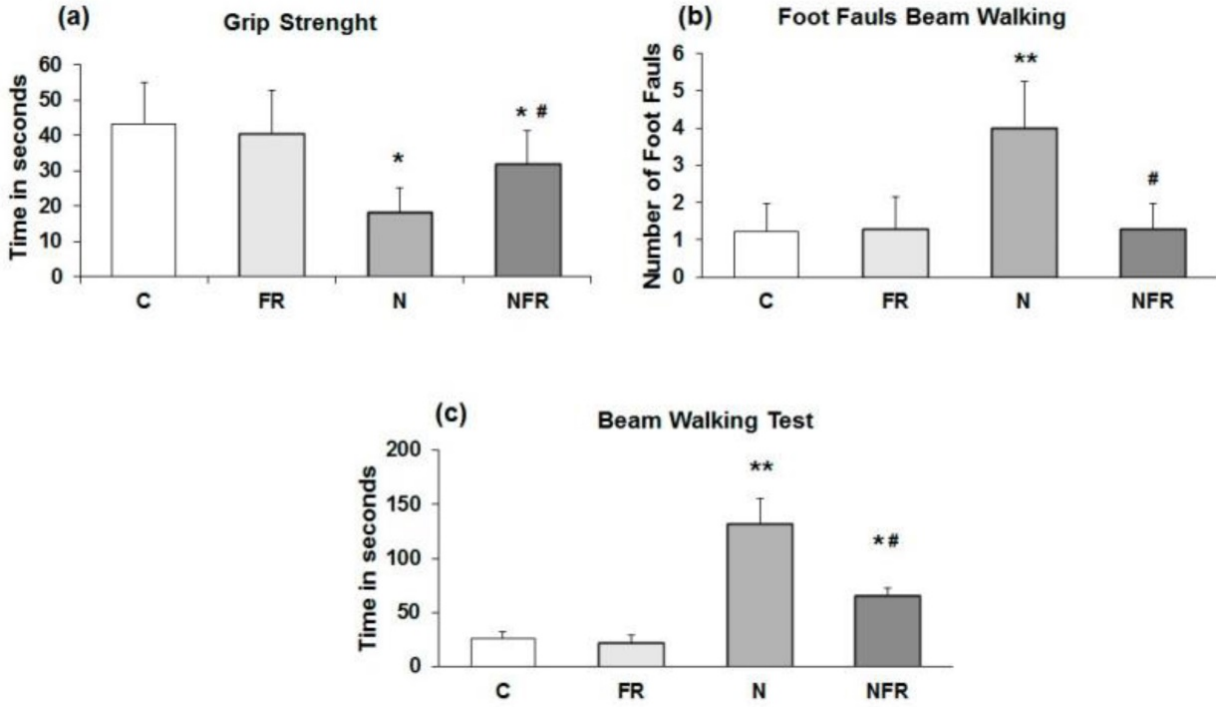
Rats without notexin (control, C and foam rolling, FR) or with notexin (notexin, N and notexin with foam rolling, NFR) were tested. (a) Grip strength (time in 60 seconds). (b) Number of foot faults. (c) Time to cross the beam (sec). Data are shown as the median for the group with error bars indicting the 95% confidence interval. **p<0.01 vs. control rats. #p < 0.05 vs. notexin.

The rats' ability to pass along a narrow beam showed using the beam walking test. C and FR did not display any general motor and muscular impairment, as demonstrated by unaffected grip strength and toe hold ability when crossing a beam in the beam walking test (Figure 1). In the grip strength test, N rats and NFR rats presented a significant time reduction compared to C rats, but NFR rats showed a longer test time than the N group (Figure 1a). Furthermore, a significant number of foot faults in the beam-walking test was observed in N rats compared to C rats (p<0.01) (Figure 1b) and a lower number of foot faults was detected in NFR rats than in N rats (p<0.01). With respect to the beam walking test, N rats took significantly longer to cross the beam than the C or foam rolling groups. NFR rats took less time than N rats (Figure 1c).

### Expression of apoptosis proteins

**Figure 2:**
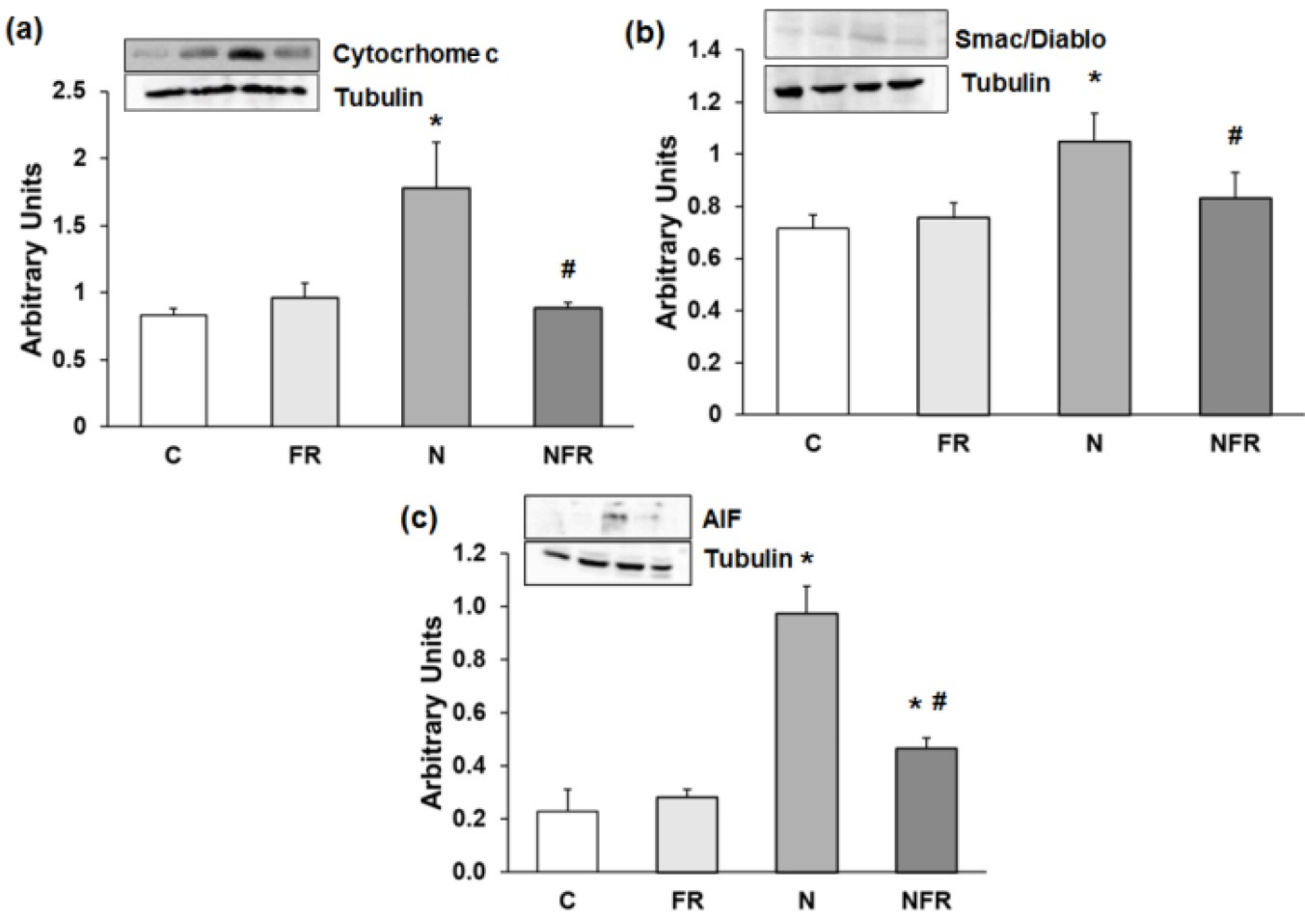
Rat muscle without notexin (control, C and foam rolling, FR) or with notexin (notexin, N and notexin with foam rolling, NFR) was collected to determine (**a**) Cytochrome c, (**b**) Smac/Diablo, and (**c**) AIF. Data are mean ± SD of six independent experiments each. Data are shown as the median for the group with error bars indicting the 95% confidence interval (N=5). *p <0.05 vs. control. #p < 0.05 vs. notexin.

Alterations in motor coordination may be related to inflammation and oxidative stress in N rats. Thus, we subsequently determined apoptosis protein expression by western blot. Our results showed that expression of Cytochrome c was significantly higher in N rats than in C rats (p<0.05) (Figure 2a). Also, significantly greater expression of another caspase-dependent apoptosis protein, Smac/Diablo, was detected in N rats than in C and foam rolling rats (p<0.05) (Figure 2b). Regarding caspase-independent proteins, such as AIF (apoptosis-inducing factor), there was significantly greater protein expression in N and NFR than in C and FR rats (p<0.05) (Figure 2c). However, foam rolling after notexin injection decreased significantly all apoptosis protein expression compared to N rats (p<0.05). Therefore, a significantly increase in apoptosis was observed after notexin injection; with a subsequent significantly decrease in apoptosis after foam rolling treatment.

### Inflammatory mediators in plasma

**Figure 3:**
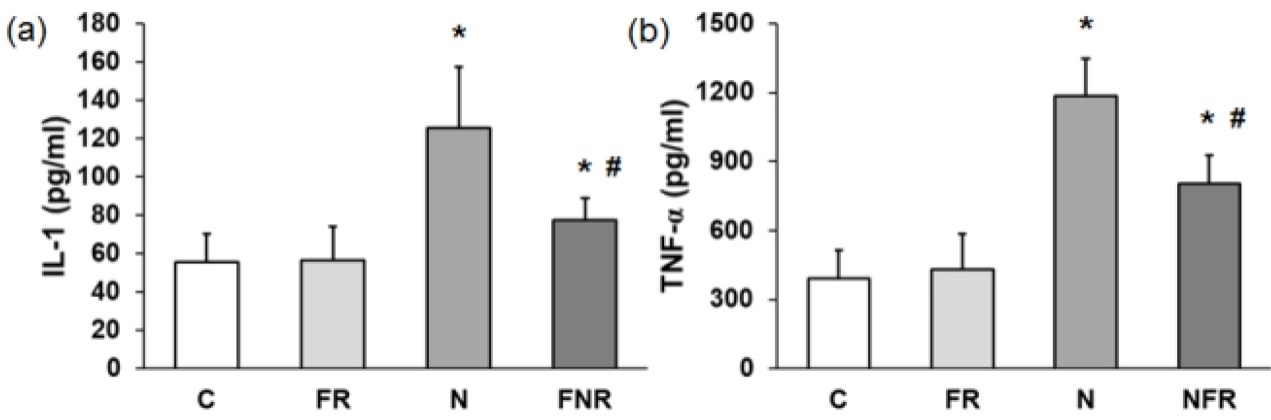
Plasma from rats without notexin (control, C and foam rolling, FR) or with notexin (notexin, N and notexin with foam rolling, NFR) was collected and (**a**) IL-1β and (**b**) TNF-α secretions were determined by ELISA. Data are shown as the median for the group with error bars indicting the 95% confidence interval (N=5). *p<0.05 vs. control. #p < 0.05 vs. notexin.

IL-1β and TNF-α pro-inflammatory proteins. Serum from the rats (C, FR, N and NFR) was assayed to determine the cytokine IL-1β and the mediator TNF-α by ELISA. Figure 3 shows that there was a 2.2-fold increase in IL-1β and a 3.5-fold increase in TNF-α in the rats treated with notexin compared to the C or foam rolling groups. However, NFR decreased IL-1β and TNF-α levels significantly compared with N values. These data indicate a decrease in notexin-induced inflammation after treatment with the foam roller (p<0.05) (Figure 3).

### Effect of foam rolling on NF-κB and IκB protein expression

**Figure 4:**
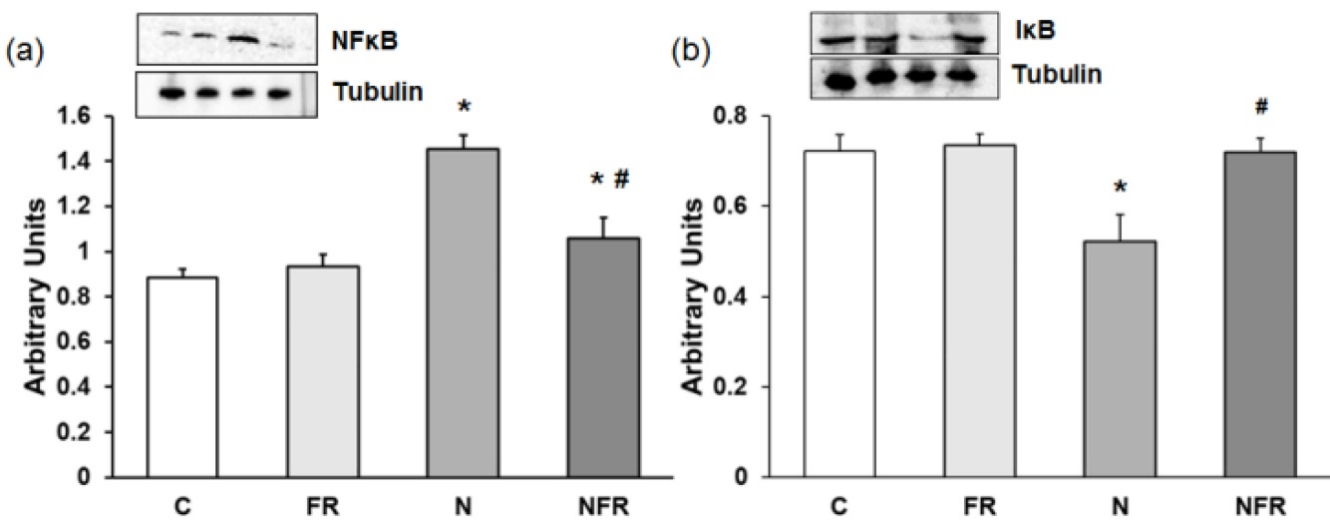
Rat muscle without notexin (control, C and foam rolling, FR) or with notexin (notexin, N and notexin with foam rolling, NFR) was collected to determine (**a**) NF-κB and (**b**) IᴋB protein expression. Data are shown as the median for the group with error bars indicting the 95% confidence interval (N=5).*p < 0.05 vs. control. #p < 0.05 vs. notexin.

We noted a significantly increase in NF-κB expression in the N group compared with the C and foam rolling groups (p<0.05) (Figure 4a). The NFR group, on the other hand, presented a significant decrease with respect to N rats (p<0.05). However, a significantly decrease in the inhibitor IκB was detected in N rats compared with C and FR rats (p<0.05). Furthermore, expression of this inhibitory factor was recovered after foam rolling was used (Figure 4b).

### COX-2 and PPAR-γ protein expression

**Figure 5:**
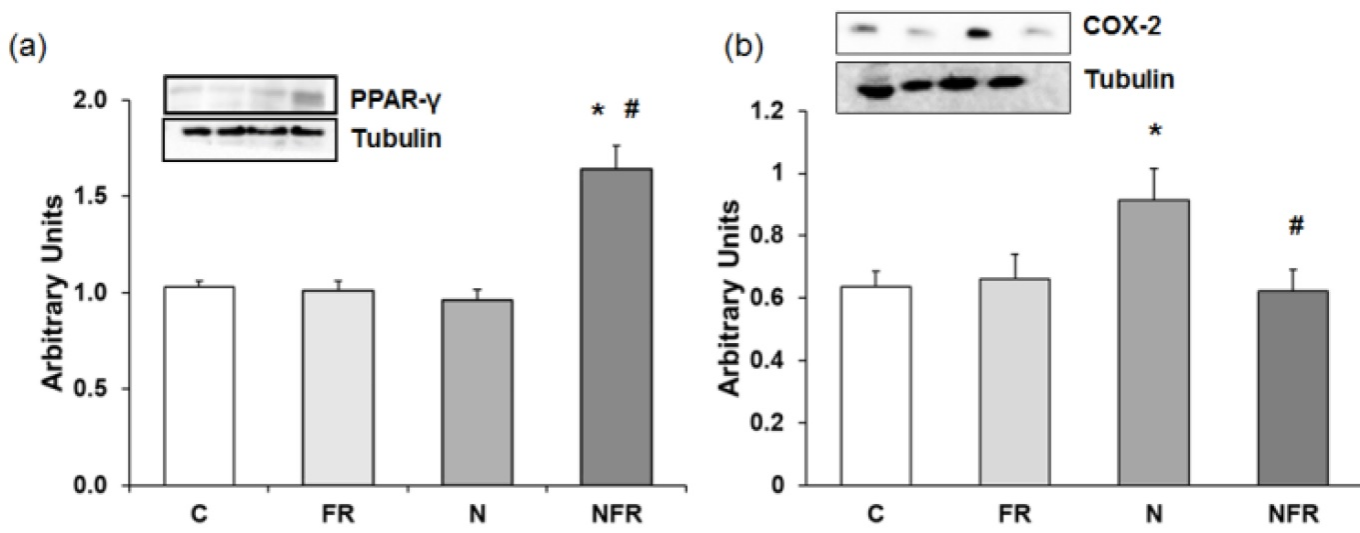
Rat muscle without notexin (control, C and foam rolling, FR) or with notexin (notexin, N and notexin with foam rolling, NFR) was collected to determine (**a**) PPAR-γ and (**b**) COX-2 protein expression. Data are shown as the median for the group with error bars indicting the 95% confidence interval (N=5).*p < 0.05 vs. control. #p < 0.05 vs. notexin.

Anti-inflammatory proteins, such as PPAR-γ (peroxisome proliferator-activated receptor γ), down-regulate gene expression of pro-inflammatory proteins. Figure 5 shows PPAR-γ expression in the different group of rats. There was a 1.5-fold increase in PPAR-γ expression in NFR rats compared to C and FR rats (Figure 5a). When notexin was injected, no changes in PPAR-γ expression were detected compared to C, indicating no activation of that anti-inflammatory protein. Also, in Figure 5b we show that N rats presented a significant increase in the inflammatory protein COX-2 compared to C and FR rats (p<0.05). Furthermore, treatment with foam rolling after notexin injection reduced significantly the expression of that pro-inflammatory protein.

### Changes in p38, p-p38 and STAT protein expression

**Figure 6:**
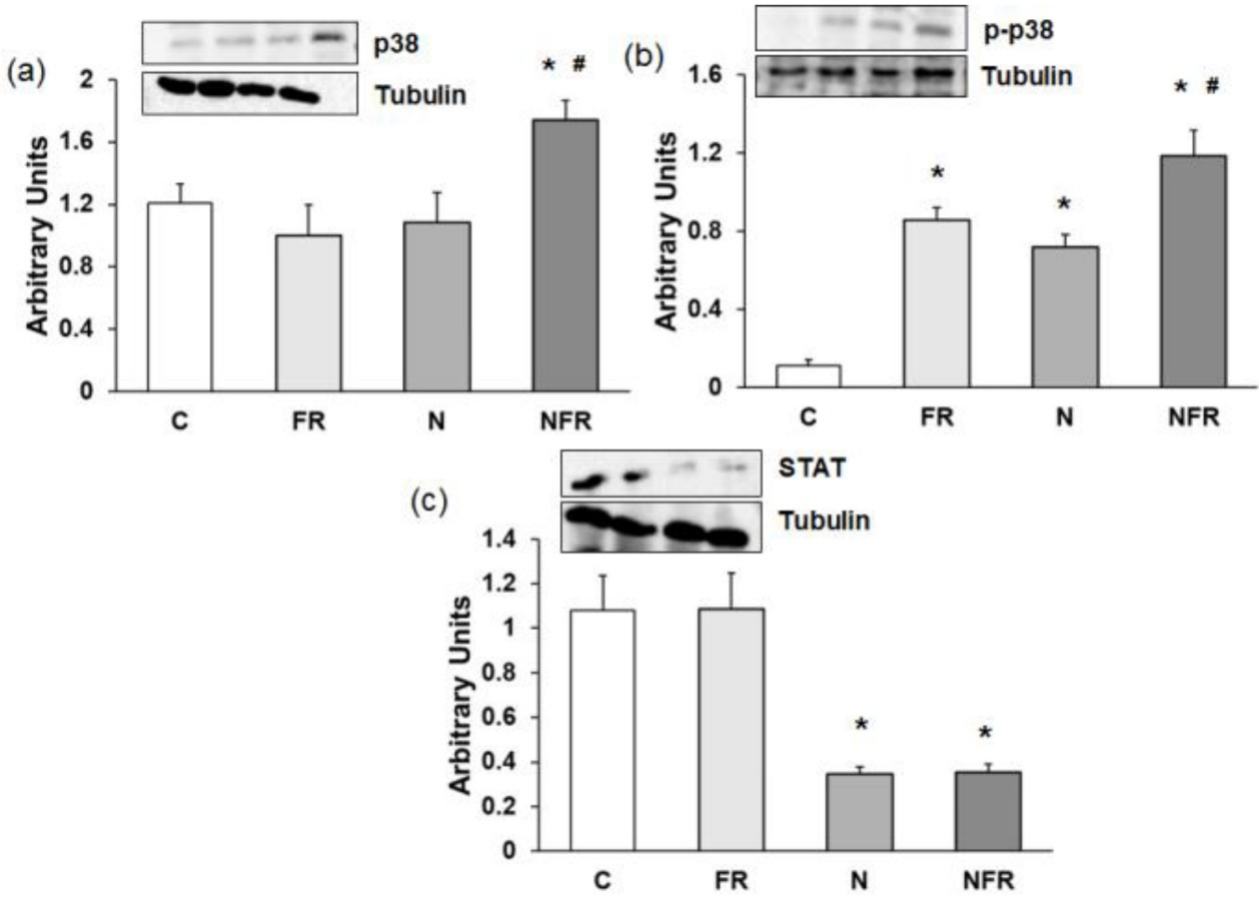
Rat muscle without notexin (control, C and foam rolling, FR) or with notexin (notexin, N and notexin with foam rolling, NFR) was collected to determine (**a**) p38, (**b**) p-p38 and (**c**) STAT protein expression. Data are shown as the median for the group with error bars indicting the 95% confidence interval (N=5).*p < 0.05 vs. control. #p < 0.05 vs. notexin.

An increase in p38 and p-p38 expression is related to an increase in cell differentiation and release of cytokines for cell-to-cell communication. Here, a significantly increase in both proteins was detected in the NFR group compared to C and FR rats (p<0.05) (Figure 6a and 6b), demonstrating an increase in cell differentiation, allowing the involvement of more muscle cells in their working functions. Otherwise no significant differences were detected between the N and NFR groups, whereas significant differences were detected between these groups and the C and FR groups. Furthermore, an inhibition of STAT protein expression improves skeletal muscle regeneration, as we detected in the N and NFR group compared to C rats (p<0.05) (Figure 6c).

### Expression of VEGF

**Figure 7:**
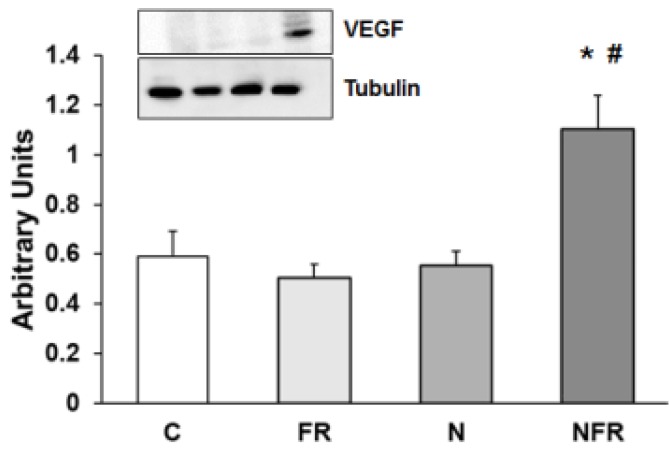
Rat muscle without notexin (control, C and foam rolling, FR) or with notexin (notexin, N and notexin with foam rolling, NFR) was collected to determine VEGF protein expression. Data are shown as the median for the group with error bars indicting the 95% confidence interval (N=5).*p < 0.05 vs. control. #p < 0.05 vs. notexin.

Vascular endothelial growth factor (VEGF) protein expression was assayed to determine vascular endothelial changes in rat quadriceps. No changes in VEGF protein expression were detected in the C, FR and N groups. However, a significantly increase in VEGF expression by applying foam rolling after the notexin injection demonstrated a rise in endothelial growth factors, producing an improvement in the vascular muscle (p<0.05) (Figure 7).

## Discussion

The present study aims to demonstrate the physiological changes produced by the use of foam rolling. To our knowledge, this is the first study that makes it possible to justify the biomolecular effects on recovery and performance after applying this technique. Under the hypothesis that foam rolling could act as a protective technique, we studied its effects on rat muscle, looking at inflammation and changes in cell death.

So far, the application of foam rolling in humans has been shown not to have a negative effect on performance^16^,^17^, and it can be used to improve recovery after applying a muscle damage induction protocol^8^,^17^. In our study we have verified that the treatment with foam rolling is capable of reversing the inflammatory process developed after the administration of notexin. So, foam rolling decreases the release of pro-inflammatory mediators induced by notexine, such as IL-1β and TNF-α, transcription factors, NF-ᴋB, and pro-inflammatory COX-2 protein. Furthermore, foam rolling also increases anti-inflammatory PPAR-γ, demonstrating protection against inflammation and probably an improvement in recovery after damage. With regard to changes in apoptosis, we detected a reduction in the expression of Smac/Diablo, Cytochrome c and AIF, demonstrating a decrease in the induction of apoptosis and probably an increase in recovery after foam roller use. The notexin action was demonstrated in melanoma and neuroblastoma cells^18^.

We noted a destruction of the cell nucleus, apoptosis and necrosis compared with untreated cells. The signalling of p38 is necessary to protect cells against apoptosis and our results demonstrated a reduction in that kinase after the addition of notexin, with a significant increase after foam roller use, thus showing muscle protection after foam roller use. However, in the present study the muscle damage caused by physical exercise has not been assessed, therefore we cannot compare the damage induced by notexin with that induced by physical exercise.

The exact mechanisms by which foam rolling leads to recovery from muscle damage remain to be determined, but under our conditions the foam roller caused a decrease in cell death due to apoptosis. The process of apoptosis is associated with the release of caspase-dependent proteins, such as Cytochrome c and Smac/Diablo, and AIF^19^, from mitochondria into the cytosol in response to cell damage. Apoptosis requires regulation of specific genes to tightly coordinate the apoptotic events^20^. The balance between cell survival and cell death is important in the mitosis process and apoptosis is very important for monitoring this.

Smac/Diablo produces apoptosis by neutralizing one or more members of the IAP (inhibitor of apoptosis proteins) family^21^. Our results demonstrate a decrease in Smac/Diablo protein expression after the use of foam rolling in muscle compared to control cells. Accordingly, the decrease after foam rolling would be associated with the inactivation of the mitochondrial apoptosis pathway^22^. It is therefore possible that the decrease in cell death would be associated with the inactivation of the mitochondrial apoptosis pathway.

In normal conditions, there is a balance of pro- and anti-inflammatory mediators in order to maintain equilibrium^22^. The majority of cytokines and protein mediators regulate cellular functions, including cell survival, growth and differentiation^23^. Our study demonstrates that foam rolling decreases the release of pro-inflammatory cytokines IL-1β and TNF-α in rat quadriceps. Notexin can produce muscle damage by various inflammatory mediators, such as IL-1β and TNF-α^24^. Prolonged or uncontrolled inflammation is detrimental, exacerbating muscle damage by over-expression of pro-inflammatory factors that enhance inflammation through a positive feedback loop, inducing the production of more cytokines or reactive oxygen species (ROS), among other deleterious effects^25^. In the first days of tissue injury, an increase in the TNF-α plasma levels has been described^26^ and it remains elevated due to its action on cellular necrosis^27^. By redox-dependent and independent pathways, TNF-α accelerates protein degradation, which promotes cell catabolism^2^, disrupts the differentiation process, and inhibits myogenesis^28^. TNF-α could stimulate catabolism, perhaps being the first potential mechanism that acts on the catabolic effect, probably by inhibiting the differentiation of myoblasts and producing the regenerative response of satellite cells to muscle damage^2^.

Moresi et al. have published that in a muscle cell culture TNF-α directly decreases total muscle protein, including adult fast-type myosin heavy chain^2^. Here we show an increase in the plasma level due to notexin and a normalized level after treatment with foam rolling for 3 days. Interactions between the action of TNF-α and that of interleukin-1 or interleukin-6 have also been published^29^. Notexin caused a significant increase in IL-1β compared to the C group. This cytokine has been associated with the inflammatory process^30^. Moreover, IL-1β inhibits protein synthesis in skeletal muscle^31^ and cardiac muscle^32^. IL-1β plasma levels return to normal values when foam rolling is used. In summary, foam rolling treatment reduces pro-inflammatory proteins.

In situations of inflammation, such as notexin-induced muscle damage, NF-ᴋB protein expression is up-regulated. Pro-inflammatory transcription factor nuclear factor κB (NF-κB) is formed by different subunits and located in the cell cytoplasm. In non-activated conditions it is bound to its inhibitor IκB. The release of NF-κB, after activation by pro-inflammatory mediators produces translocation to the nucleus and subsequent transcription of pro-inflammatory proteins^33^. Accordingly, we found an increase in NF-ᴋB/p-65 expression with a decrease in its inhibitor IᴋB in muscle after notexin injection. Different authors have found that the cytokine-receptor complex is able to bind to cytokines and other proteins of the extracellular matrix, producing inflammatory signals which could be important in different pathologies^34^. Foam rolling decreases the pro-inflammatory transcription factor (NF-ᴋB), reducing the production of pro-inflammatory proteins such as IL-1β, TNF-α and COX-2^35^.

Inducible cyclooxygenase (COX-2) and the involvement of neurotrophic factors have been related to delayed-onset muscle soreness^36^, as we found after notexin injection. Foam rolling would be a better method for recovery from muscle damage rather than non-steroidal anti-inflammatory drugs and toxins^37^.

It has been shown in the literature that notexin produces inactivation of the MAP kinase cascade^38^, affecting the p38 MAPK pathway. Increases in p38 and p-p38 expression are related to an increase in cell differentiation and the release of cytokines for cell-to-cell communication^39^. Myoblast differentiation is complex, with a specific transcriptional program that exits the cell cycle and changes the cell's morphology, producing elongation, alignment, and creating syncytial myofibres. The transcription factors produced are regulated by specific signal transduction pathways, including the p38 MAP kinase pathway^40^. We detected that foam rolling increased p38 and p-p38, showing an induction of that kinase to protect against notexin-induced damage and increasing myoblast differentiation. Here, an increase in both proteins was detected in the NFR group compared to C and FR rats, demonstrating an increase in cell differentiation, allowing the involvement of more muscle cells in their working functions. Furthermore, an inhibition of STAT protein improves skeletal muscle regeneration, as we detected after foam rolling was used in N rats. Changes in signalling after foam roller use in terms of regeneration and differentiation lead to improved health after notexin-induced damage.

Because of the possible protective mechanism against inflammation after foam rolling, a decrease in pro-inflammatory and an increase in anti-inflammatory proteins may be activated by foam rolling. Indeed, PPAR-γ plays a fundamental role in the immune response because it decreases the expression of pro-inflammatory genes^40^, also acting as an anti-inflammatory protein, and suppresses NF-κB^41^. Recent data suggest that the NF-κB inhibitory pathway may stimulate the N2 neutrophil phenotype, with neuroprotective effects^42^. In addition, PPAR-γ increases fatty acid uptake and esterification, promoting myocellular lipid storage, simultaneously increasing insulin signalling and glycogen formation. All of these have positive effects on metabolic health and tissue repair. Our results demonstrate that notexin produces a significant decrease in PPAR-γ expression compared to C rats. Foam rolling treatment significantly increases that anti- inflammatory protein reduced by notexin and returns it to control values. Changes in apoptosis and inflammation after the application of foam rolling could justify the attenuation of perceived muscle pain in healthy adults after muscle damage^5^. This decrease in perceived muscle pain could be related to an improvement in muscle recovery, which could lead to better performance as various authors have shown^9^,^43^.

On the other hand, vascular endothelial growth factor (VEGF) is a paracrine factor that plays a major role in the promotion of angiogenesis, improving cellular survival, inducing proliferation and increasing the migration and invasion of endothelial cells. VEGF is also induced by PPAR-γ in cardiac myofibroblasts^44^, diminishing inflammation. During contraction skeletal muscle fibres control capillary growth by releasing VEGF^45^. Also, VEGF produces skeletal muscle regeneration by stimulating myogenic differentiation through stem cells^46^. Our results demonstrate a high degree of VEGF protein expression after notexin damage in the NFR group. Hotfiel et al. found that blood flow increased significantly after foam rolling exercises in healthy participants^11^. Moreover, the compression caused by foam rolling over blood vessels reduces arterial stiffness and improves vascular endothelial function^11^. This could be due to a distortion of the vascular endothelium, which could induce the release of nitric oxide (NO)^47^.

**Figure 8:**
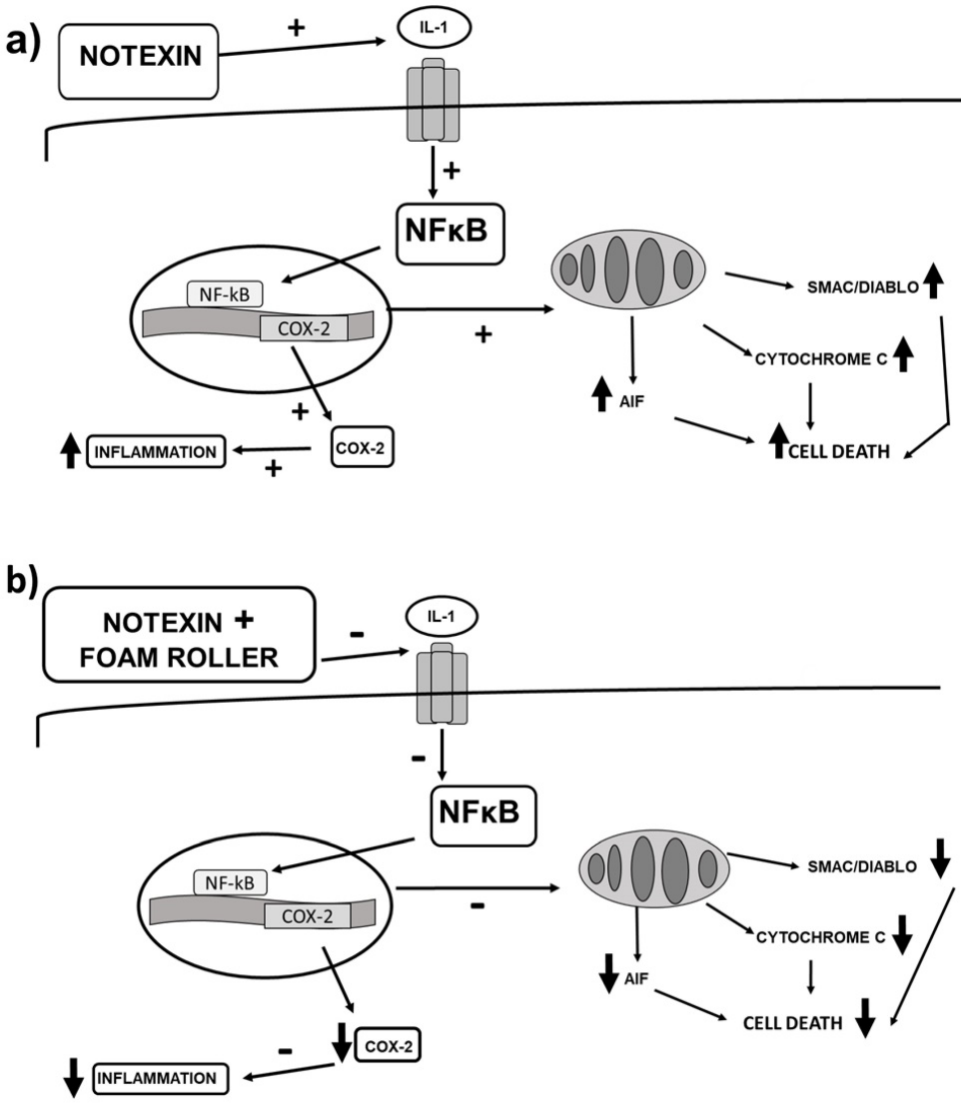
(**a**) Signalling changes in inflammation and cell death after notexin-induced muscle damage in rats, and (**b**) changes that foam rolling can trigger after notexin-induced muscle damage. Foam rolling might protect muscle cells from inflammation and cell death.

In summary, our study demonstrates an increase in apoptosis and inflammation after notexin injection (Figure 8a) and considerable improvements after the application of foam rolling (Figure 8b). This last figure shows the changes that foam rolling can have after notexin-induced muscle damage. Based on this figure (Figure 8b), it can be stated that this is the first research on the effect of foam rolling on muscle tissue that shows the biomolecular mechanisms triggered. This experimental work provides a basis for clinical trials to be carried out to confirm the effectiveness of foam rolling in humans.

## Conclusions

This study demonstrates that foam rolling prevents the increase in Cytochrome c, AIF and Smac/Diablo expression induced by notexin in quadriceps muscles, indicating that foam rolling prevents notexin-induced cell death by apoptosis. Furthermore, a reduction in pro-inflammatory mediators (NF-κB, IL-1β, TNF-α and COX-2) and increase in anti-inflammatory mediators (PPAR-γ) can result in recovery from notexin-induced damage. Moreover, an increase in VEGF was detected. These results would justify the use of foam rolling as a method for muscle recovery after toxic damage.

## References

[1] Terra R, da Silva SAG, Pinto VS, Dutra PML (2012). Effect of exercise on immune system: response, adaptation and cell signaling. Rev Bras Med Esporte.

[2] Moresi V, Pristerà A, Scicchitano BM, Molinaro M, Teodori L, Sassoon D, Adamo S, Coletti, D (2008). Tumor Necrosis Factor-α Inhibition of Skeletal Muscle Regeneration Is Mediated by a Caspase-Dependent Stem Cell Response. Stem Cells.

[3] Liu CS, Chang CC, Du YC, Chang FR, Wu YC, Chang WC, Hsieh TJ (2012). 2-hydroxy-4'-methoxychalcone inhibits proliferation and inflammation of human aortic smooth muscle cells by increasing the expression of peroxisome proliferator-activated receptor gamma. J Cardiovasc Pharmacol.

[4] Thom R, Rowe GC, Jang C, Safdar A, Arany Z (2014). Hypoxic induction of vascular endothelial growth factor (VEGF) and angiogenesis in muscle by truncated peroxisome proliferator-activated receptor γ coactivator (PGC)-1α. J Biol Chem.

[5] Fullerton JN, Gilroy DW (2016). Resolution of inflammation: a new therapeutic frontier. Nat Rev Drug Discov.

[6] Macdonald GZ, Button DC, Drinkwater EJ, Behm DG (2014). Foam Rolling as a Recovery Tool after an Intense Bout of Physical Activity. Med Sci Sports Exerc.

[7] Cheatham SW, Kolber MJ, Cain M, Lee M (2015). The effects of self-myofascial release using a foam roll or roller massager on joint range of motion, muscle recovery, and performance: a systematic review. Int J Sports Phys Ther.

[8] Romero-Moraleda B, Gonzalez-Garcia J, Cuellar-Rayo A, Balsalobre-Fernandez C, Munoz-Garcia D, Morencos E (2019). Effects of Vibration and Non-Vibration Foam Rolling on Recovery after Exercise with Induced Muscle Damage. J Sports Sci Med.

[9] Rey E, Padrón-Cabo A, Costa PB, Barcala-Furelos R (2019). The Effects of Foam Rolling as a Recovery Tool in Professional Soccer Players. J Strength Cond Res.

[10] Okamoto T, Masuhara M, Ikuta K (2014). Acute effects of self-myofascial release using a foam roller on arterial function. J Strength Cond Res Natl Strength Cond Assoc.

[11] Hotfiel T, Swoboda B, Krinner S, Grim C, Engelhardt M, Uder M, Heiss RU (2017). Acute Effects of Lateral Thigh Foam Rolling on Arterial Tissue Perfusion Determined by Spectral Doppler and Power Doppler Ultrasound. J Strength Cond Res.

[12] Lohman EB, Bains GS, Lohman T, DeLeon M, Petrofsky JS (2011). A comparison of the effect of a variety of thermal and vibratory modalities on skin temperature and blood flow in healthy volunteers. Med Sci Monit Int Med J Exp Clin Res.

[13] Plant DR, Colarossi FE, Lynch GS (2006). Notexin causes greater myotoxic damage and slower functional repair in mouse skeletal muscles than bupivacaine. Muscle Nerve.

[14] Peterson GL (1977). A simplification of the protein assay method of Lowry et al. which is more generally applicable. Anal Biochem.

[15] Hunter A, Hatcher J, Virley D, Nelson P, Irving E, Hadingham S, Parsons AA (2000). Functional assessments in mice and rats after focal stroke. Neuropharmacology.

[16] Behara B, Jacobson BH (2017). The Acute Effects of Deep Tissue Foam Rolling and Dynamic Stretching on Muscular Strength, Power, and Flexibility in Division I Linemen. J Orthop Trauma.

[17] D'Amico AP, Gillis J (2019). The influence of foam rolling on recovery from exercise-induced muscle damage. J Strength Cond Res.

[18] Khunsap S, Khow O, Buranapraditkun S, Suntrarachun S, Puthong S, Boonchang S (2016). Anticancer properties of phospholipase A2 from Daboia siamensis venom on human skin melanoma cells. J Venom Anim Toxins Trop Dis.

[19] Du C, Fang M, Li Y, Li L, Wang X (2000). Smac, a Mitochondrial Protein that Promotes Cytochrome c-Dependent Caspase Activation by Eliminating IAP Inhibition. Cell.

[20] Danial NN, Korsmeyer SJ (2004). Cell death: critical control points. Cell.

[21] Tchoghandjian A, Soubéran A, Tabouret E, Colin C, Denicolaï E, Jiguet-Jiglaire C, El-Battari A, Villard C, Baeza-Kallee N, Figarella-Branger D (2016). Inhibitor of apoptosis protein expression in glioblastomas and their in vitro and in vivo targeting by SMAC mimetic GDC-0152. Cell Death Dis.

[22] Lee M-S (2016). Role of mitochondrial function in cell death and body metabolism. Front Biosci Landmark Ed.

[23] Holdsworth SR, Gan P-Y (2015). Cytokines: Names and Numbers You Should Care About. Clin J Am Soc Nephrol.

[24] Yang W, Hu P (2018). Skeletal muscle regeneration is modulated by inflammation. J Orthop Transl.

[25] Swiderski K, Thakur SS, Naim T, Trieu J, Chee A, Stapleton DI, Koopman R, Lynch GS (2016). Muscle-specific deletion of SOCS3 increases the early inflammatory response but does not affect regeneration after myotoxic injury. Skelet Muscle.

[26] Jabari D, Vedanarayanan VV, Barohn RJ, Dimachkie MM (2018). Update on Inclusion Body Myositis. Curr Rheumatol Rep.

[27] Meador BM, Krzyszton CP, Johnson RW, Huey KA (2008). Effects of IL-10 and age on IL-6, IL-1β, and TNF-α responses in mouse skeletal and cardiac muscle to an acute inflammatory insult. J Appl Physiol.

[28] Bhatnagar S, Panguluri SK, Gupta SK, Dahiya S, Lundy RF, Kumar A (2010). Tumor Necrosis Factor-α Regulates Distinct Molecular Pathways and Gene Networks in Cultured Skeletal Muscle Cells. PLoS ONE.

[29] Langen RCJ, Van Der Velden JLJ, Schols AMWJ, Kelders MCJM, Wouters EFM, Janssen-Heininger YMW (2004). Tumor necrosis factor-alpha inhibits myogenic differentiation through MyoD protein destabilization. FASEB J Off Publ Fed Am Soc Exp Biol.

[30] Shi F, Xiong Y, Zhang Y, Qiu C, Li M, Shan A, Yang Y, Li B (2018). The Role of TNF Family Molecules Light in Cellular Interaction Between Airway Smooth Muscle Cells and T Cells During Chronic Allergic Inflammation. Inflammation.

[31] Roerink ME, Bredie SJH, Heijnen M, Dinarello CA, Knoop H, Van der Meer JWM (2017). Cytokine Inhibition in Patients With Chronic Fatigue Syndrome: A Randomized Trial. Ann Intern Med.

[32] Borghi SM, Zarpelon AC, Pinho-Ribeiro FA, Cardoso RDR, Cunha TM, Alves-Filho JC, Ferreira SH, Cunha FQ, Casagrande R, Verri WA Jr (2014). Targeting interleukin-1β reduces intense acute swimming-induced muscle mechanical hyperalgesia in mice: IL-1β mediates postexercise muscle pain. J Pharm Pharmacol.

[33] Aguirre-Rueda D, Guerra-Ojeda S, Aldasoro M, Iradi A, Obrador E, Mauricio MD, Vila JM, Marchio P, Valles SL (2015). WIN 55,212-2, Agonist of Cannabinoid Receptors, Prevents Amyloid β1-42 Effects on Astrocytes in Primary Culture. PLOS ONE.

[34] Yang Z, Kirton HM, Al-Owais M, Thireau J, Richard S, Peers C, Steele DS (2017). Epac2-Rap1 Signaling Regulates Reactive Oxygen Species Production and Susceptibility to Cardiac Arrhythmias. Antioxid Redox Signal.

[35] Bondareff W (2013). Age-related changes in brain extracellular space affect processing of amyloid-β peptides in Alzheimer's disease. J Alzheimers Dis JAD.

[36] Mizumura K, Taguchi T (2016). Delayed onset muscle soreness: Involvement of neurotrophic factors. J Physiol Sci.

[37] Fleming JA, Naughton RJ, Harper LD (2018). Investigating the Nutritional and Recovery Habits of Tennis Players. Nutrients.

[38] Chen K-C, Chang L-S (2010). Notexin upregulates Fas and FasL protein expression of human neuroblastoma SK-N-SH cells through p38 MAPK/ATF-2 and JNK/c-Jun pathways. Toxicon.

[39] Krauss RS (2017). Regulation of Skeletal Myoblast Differentiation by Drebrin. Adv Exp Med Biol.

[40] Bertin B, Dubuquoy L, Colombel J-F, Desreumaux P (2013). PPAR-gamma in ulcerative colitis: a novel target for intervention. Curr Drug Targets.

[41] Wan Y, Chong L-W, Evans RM (2007). PPAR-γ regulates osteoclastogenesis in mice. Nat Med.

[42] Cuartero MI, Ballesteros I, Moraga A, Nombela F, Vivancos J, Hamilton JA, Corbí ÁL, Lizasoain I, Moro MA (2013). N2 Neutrophils, Novel Players in Brain Inflammation After Stroke: Modulation by the PPARγ Agonist Rosiglitazone. Stroke.

[43] Fleckenstein J, Wilke J, Vogt L, Banzer W (2017). Preventive and Regenerative Foam Rolling are Equally Effective in Reducing Fatigue-Related Impairments of Muscle Function following Exercise. J Sports Sci Med.

[44] Chintalgattu V, Harris GS, Akula SM, Katwa LC (2007). PPAR-gamma agonists induce the expression of VEGF and its receptors in cultured cardiac myofibroblasts. Cardiovasc Res.

[45] Hoier B, Prats C, Qvortrup K, Pilegaard H, Bangsbo J, Hellsten Y (2013). Subcellular localization and mechanism of secretion of vascular endothelial growth factor in human skeletal muscle. FASEB J.

[46] Beckman SA, Chen WCW, Tang Y, Proto JD, Mlakar L, Wang B, Huard J (2013). Beneficial Effect of Mechanical Stimulation on the Regenerative Potential of Muscle-Derived Stem Cells Is Lost by Inhibiting Vascular Endothelial Growth Factor. Arterioscler Thromb Vasc Biol.

[47] Huey KA Potential Roles of Vascular Endothelial Growth Factor During Skeletal Muscle Hypertrophy: Exerc Sport Sci Rev. 2018; 46: 195-202. DOI: 10.1249/JES.0000000000000152.

